# State Substitution Laws and Uptake of an Interchangeable Insulin Biosimilar

**DOI:** 10.1001/jamahealthforum.2025.0406

**Published:** 2025-04-04

**Authors:** Youngmin Kwon, Ameet Sarpatwari, Stacie B. Dusetzina

**Affiliations:** 1 Department of Health Policy, Vanderbilt University Medical Center, Nashville, Tennessee; 2 Division of Health Policy and Insurance Research, Department of Population Medicine, Harvard Pilgrim Health Care Institute and Harvard Medical School, Boston, Massachusetts

## Abstract

**Importance:**

Although biosimilars have potential to reduce drug spending, their use remains low. Automatic substitutions of biologic drugs by their biosimilars at pharmacies can facilitate biosimilar uptake. Yet, state regulations limiting the types and circumstances under which biosimilars may be substituted could discourage efficient biosimilar adoption.

**Objective:**

To examine associations of state substitution laws in the US with biosimilar adoption in the insulin glargine market, in which an interchangeable biosimilar was recently launched.

**Design, Setting, and Participants:**

This retrospective cohort study using MarketScan commercial claims data examined fills for insulin glargine among users who were younger than 65 years and had fills for the insulin glargine Lantus (Sanofi) and its biosimilars, interchangeable insulin glargine-yfgn (Semglee [Mylan Pharmaceuticals]) and a noninterchangeable insulin glargine (Basaglar [Lilly]). Data were analyzed from August 2024 to January 2025.

**Main Outcomes and Measures:**

Market share of the insulin glargine, interchangeable insulin glargine-yfgn, and the noninterchangeable insulin glargine. Unit of analysis was the per-person prescription fill. Changes in fills between those residing in states with less vs more restrictive substitution laws in a 1-year period before and after the launch of interchangeable glargine-yfgn (November 16, 2021) were compared.

**Results:**

A total of 487 281 per-person prescription fills (mean [SD] age, 49.5 [13.3] years; 56.9% male) were included, with 158 141 and 329 140 per-person prescription fills from less vs more restrictive states, respectively. Following the launch of insulin glargine-yfgn, its market share differentially increased by 7.03 percentage points (pp; 95% CI, 1.89-12.18 pp; *P* = .008), coinciding with a 6.48 pp (95% CI, −11.70 to −1.26 pp; *P* = .02) differential reduction in the insulin glargine market share in states with less vs more restrictive laws. In the last quarter, the market share for insulin glargine-yfgn was 20.6% and 12.1% in states with less and more restrictive laws, respectively. There were not statistically significant differential changes in fills for the noninterchangeable insulin glargine (−0.24 pp; 95% CI, −1.40 to 0.92 pp; *P* = .68). Three restrictions had more pronounced associations with a lower uptake of insulin glargine-yfgn: enhanced physician notification (−8.15 pp; 95% CI, −12.49 to −3.81 pp; *P* < .001), refill notifications (−4.68 pp; 95% CI, −8.78 to −0.58 pp; *P* = .03), and patient notification (−3.52 pp; 95% CI, −8.44 to 1.40 pp; *P* = .16).

**Conclusions and Relevance:**

In this cohort study, insulin users in states with less restrictive substitution laws were more likely to fill the biosimilar alternative to insulin glargine, underscoring the role of state regulations of substitution as an important determinant of biosimilar adoption and the need for reforms to increase efficient biosimilar adoption.

## Key Points

**Question:**

Are state laws in the US regulating pharmacist substitution of branded biologics with biosimilars associated with biosimilar adoption among commercially insured individuals receiving a long-acting insulin glargine product with an interchangeable biosimilar?

**Findings:**

In this cohort study of 487 281 long-acting insulin fills, commercially insured insulin users in states with less stringent substitution laws were 7.03 percentage points more likely to fill prescriptions for the interchangeable biosimilar following its market launch, relative to insulin users in states with more restrictive substitution laws.

**Meaning:**

State substitution laws are associated with uptake of an interchangeable insulin glargine biosimilar; reducing restrictions on substitution may facilitate expanded biosimilar use.

## Teaser

This cohort study examines associations of state substitution laws in the US with biosimilar adoption in the insulin glargine market in which an interchangeable biosimilar (Semglee) was recently launched.

## Introduction

Biosimilars have potential to lower spending on biologic drugs, but their uptake in the US has been slow.^1^ Due to their unique pharmacological properties (ie, similar, but not identical, to biologics), biosimilars can engender concerns about safety and efficacy, contributing to limited prescribing.^2^ To address this concern, Congress created a special “interchangeability” pathway for biosimilars demonstrating comparable safety and efficacy in a robust switching study.^3^ Legally, interchangeability designation is important because it confers authority for pharmacists to substitute biologic drugs at the point of dispensing. Pharmacies may favor dispensing of interchangeable biosimilars, which likely have lower acquisition costs,^4^ but they must also comply with state regulations on substitution practices that may generate administrative burden for the substituting pharmacists.^5^ In markets for generic small molecule drugs, restrictive substitution laws have been associated with lower uptake of generics, suggesting that substitution laws can be a powerful policy tool for facilitating or blocking entrance of nonbranded products.^4^ To our knowledge, no prior investigations have examined the role of substitution laws in biosimilar uptake. In this study, we estimate the associations of biosimilar substitution laws with the dispensing of a biosimilar to a long-acting insulin glargine, the first biosimilar to receive the interchangeability designation.^6^

## Methods

### Study Design

We conducted a retrospective analysis of pharmacy claims in the MarketScan commercial claims data, spanning November 2020 to November 2022 (eMethods in Supplement 1). Institutional review board approval was waived because we analyzed deidentified patient-level data that were fully compliant with HIPAA (US Health Insurance Portability and Accountability Act). We followed the Strengthening the Reporting of Observational Studies in Epidemiology (STROBE) reporting guidelines for reporting observational studies.

The study population included commercially insured enrollees who were younger than 65 years and had at least 1 fill for a highly prescribed long-acting insulin glargine (Lantus [Sanofi]) or its substitutes, interchangeable biosimilar insulin glargine-yfgn (Semglee [Mylan Pharmaceuticals]) and a noninterchangeable insulin glargine (Basaglar [Lilly]) (eTable 1 in Supplement 1), in the 12-month period before and after November 16, 2021, when interchangeable insulin glargine-yfgn was launched (insulin glargine-yfgn was initially launched as a noninterchangeable biosimilar in June 2020^6^). We included the noninterchangeable substitute, which was approved as a follow-on biologic to the highly prescribed insulin glargine, because it is widely considered a biosimilar in clinical practice.^7,8^ We excluded Toujeo (Sanofi; another branded insulin glargine) from the denominator because our focus was on understanding the extent to which state laws may influence the substitution of the highly prescribed insulin glargine with insulin glargine-yfgn (the other branded insulin glargine cannot be substituted with insulin glargine-yfgn).

We obtained state-level substitution laws from a repository of state laws (as of July 2021) compiled by the National Association of Chain Drug Stores,^9^ which we verified with online searches of public databases. We characterized 7 domains of substitution laws, each denoting a more restrictive requirement for substitution and receiving a score of 1 (Table 1). We created a summary score for each state and an exposure variable, categorizing states with less vs more restrictive laws based on the median score (≤3 vs >3; eFigures 1 and 2 in Supplement 1). We merged this variable to each fill, using its geographic identifier. For a quarter of fills, this identifier listed US Census Bureau region, rather than state (we examined sensitivity of the results to exclusion of these fills). We excluded Vermont, Delaware, and Washington, DC, because claims in 2022 were missing in these areas.

**Table 1.** Domains of Biosimilar Substitution Laws

Domain^a^	Description	No. of states (%)^b^
Active approval	Express authorization is required by prescribers for substitution	5 (10)
Enhanced physician notification	Pharmacists must notify prescribing physicians within 5 d	10 (21)
Notification for refills	No clear exemption for physician notification for refills	3 (6)
Patient notification	Patients must be notified at the time of dispensing	43 (90)
Enhanced labeling requirement	The label must include an explicit language stating substitution (such as “substituted for”)	21 (44)
Record retention	Pharmacists must retain records of substitution	23 (48)
Unspecified legal liability	No clear language stating that pharmacists assume no greater liability for dispensing biosimilar vs the branded product	31 (65)

^a^ Seven domains of substitution laws were evaluated in the study. Each domain represents a potentially more restrictive requirement for pharmacists who wish to substitute at the point of dispensing. Each domain was characterized by reviewing relevant legal codes and statutes in each state (see Methods and eMethods in Supplement 1 for more details).

^b^ The number of states with substitution laws falling within each domain (N = 48). Vermont, Delaware, and Washington, DC, were excluded from the analysis, as claims from these areas were missing in 2022.

### Statistical Analysis

We conducted a difference-in-differences analysis, comparing differential changes in market share for each product between less vs more restrictive states, following the launch of interchangeable insulin glargine-yfgn. We controlled for enrollee- and year-quarter fixed effects, adjusting for time-invariant enrollee-level characteristics and secular trends, and clustered standard errors at the state level. We also estimated differential changes in the outcome in each year-quarter using an event-study model.

We performed several robustness checks. First, we conducted a falsification test by examining changes in fills for the insulin glargine that is a noninterchangeable biosimilar for the highly prescribed insulin glargine. Because state substitution laws only permit substitution of biologic products with their interchangeable biosimilars, we expected a null change in the noninterchangeable insulin glargine’s market share. Second, we assessed patterns of fills for other long-acting insulin products (Levemir [Novo Nordisk], Toujeo, and Tresiba [Novo Nordisk]) to ensure that any changes in fills for the highly prescribed insulin glargine were attributed to substitution of the insulin glargine with insulin glargine-yfgn alone. Third, we investigated outlier states via a leave-one-out analysis. Fourth, we examined the robustness of the exposure variable by (1) using each substitution law domain as separate exposure variables and (2) excluding each domain when constructing the exposure variable.

Data were analyzed from August 2024 to January 2025 using SAS Studio (SAS Institute) and Stata, version 14 (StataCorp). We used a 2-sided *P *value with α = .05 to assess statistical significance.

## Results

A total of 487 281 per-person prescription fills were included (mean [SD] age, 49.5 [13.3] years; 56.9% male) for the highly prescribed insulin glargine, noninterchangeable insulin glargine, or insulin glargine-yfgn, with 158 141 (32.5%) and 329 140 (67.6%) from states with less and more restrictive laws, respectively. The number of fills decreased from 262 446 to 224 835 after the launch of insulin glargine-yfgn. Following the launch of insulin glargine-yfgn, there was a 7.03 percentage point (pp; 95% CI, 1.89-12.18 pp; *P* = .008) differential increase in its market share and a 6.48 pp (95% CI, −11.70 to −1.26 pp; *P* = .02) decrease in the highly prescribed insulin glargine market share in states with less vs more restrictive laws (Table 2). There were not statistically significant changes in fills for the noninterchangeable insulin glargine, which is not subjected to state substitution laws (−0.24 pp; 95% CI, −1.40 to 0.92 pp; *P* = .68). Event study plots suggest no statistically significant pretrends (Figure). In sensitivity checks, switching between long-acting insulin products was ruled out (eTables 2 and 3 in Supplement 1), as were statistically significant outlier states (eFigure 3 in Supplement 1), and exclusions of fills with missing state identifiers were confirmed to not have appreciably influenced estimates (eTable 4 and eFigure 4 in Supplement 1). When considering the specific substitution law domains, the following were more strongly associated with a lower uptake of insulin glargine-yfgn: enhanced physician notification (−8.15 pp; 95% CI, −12.49 to −3.81 pp; *P* < .001), notification for refills (−4.68 pp; 95% CI, −8.78 to −0.58 pp; *P* = .03), and patient notification (−3.52 pp; 95% CI, −8.44 to 1.40 pp; *P* = .16) (eTable 5 in Supplement 1). Results are robust to excluding each domain in the calculation of the exposure (eAppendix and eTable 6 in Supplement 1).

**Table 2.** Difference-in-Differences Estimates of Changes in Insulin Glargine Market Share Associated With State Substitution Laws^a^

Insulin glargine product	% (95% CI)				Adjusted difference in differences		Relative change, %^b^
	States with more restrictive substitution laws		States with less restrictive substitution laws				
	Before launch	After launch	Before launch	After launch	Estimate (95% CI), percentage point	*P* value	
Lantus	59.6 (4.3 to 73.9)	49.5 (36.7 to 62.3)	58.6 (53.9 to 63.2)	41.1 (35.2 to 47.0)	−6.48 (−11.70 to −1.26)	.02	−11.1
Semglee	0.1 (−0.01 to 0.2)	8.9 (6.7 to 11.0)	0.1 (0.0 to 0.2)	15.7 (11.5 to 19.8)	7.03 (1.89 to 12.18)	.008	>100
Basaglar	40.4 (26.0 to 54.7)	41.1 (27.4 to 54.8)	41.4 (36.7 to 46.1)	43.1 (37.0 to 49.2)	−0.24 (−1.40 to 0.92)	.68	−0.6

^a^ The table reports difference-in-differences estimates, showing differential changes in outcomes between insulin users filling a highly prescribed long-acting insulin glargine (Lantus [Sanofi]), interchangeable biosimilar insulin glargine-yfgn (Semglee [Mylan Pharmaceuticals]), or a noninterchangeable insulin glargine (Basaglar [Lilly]) in states with less restrictive substitution laws, defined as having a summary substitution law score of 3 or less and other states with more restrictive substitution laws, before and after the market launch date of interchangeable insulin glargine-yfgn on November 16, 2021. The unit of analysis is per-person prescription fill. Estimates adjusted for person fixed effects and year-quarter fixed effects; standard errors were clustered at the state level.

^b^ Relative change was calculated as the difference-in-differences estimates divided by the preperiod mean in states with less restrictive substitution laws.

**Figure.**
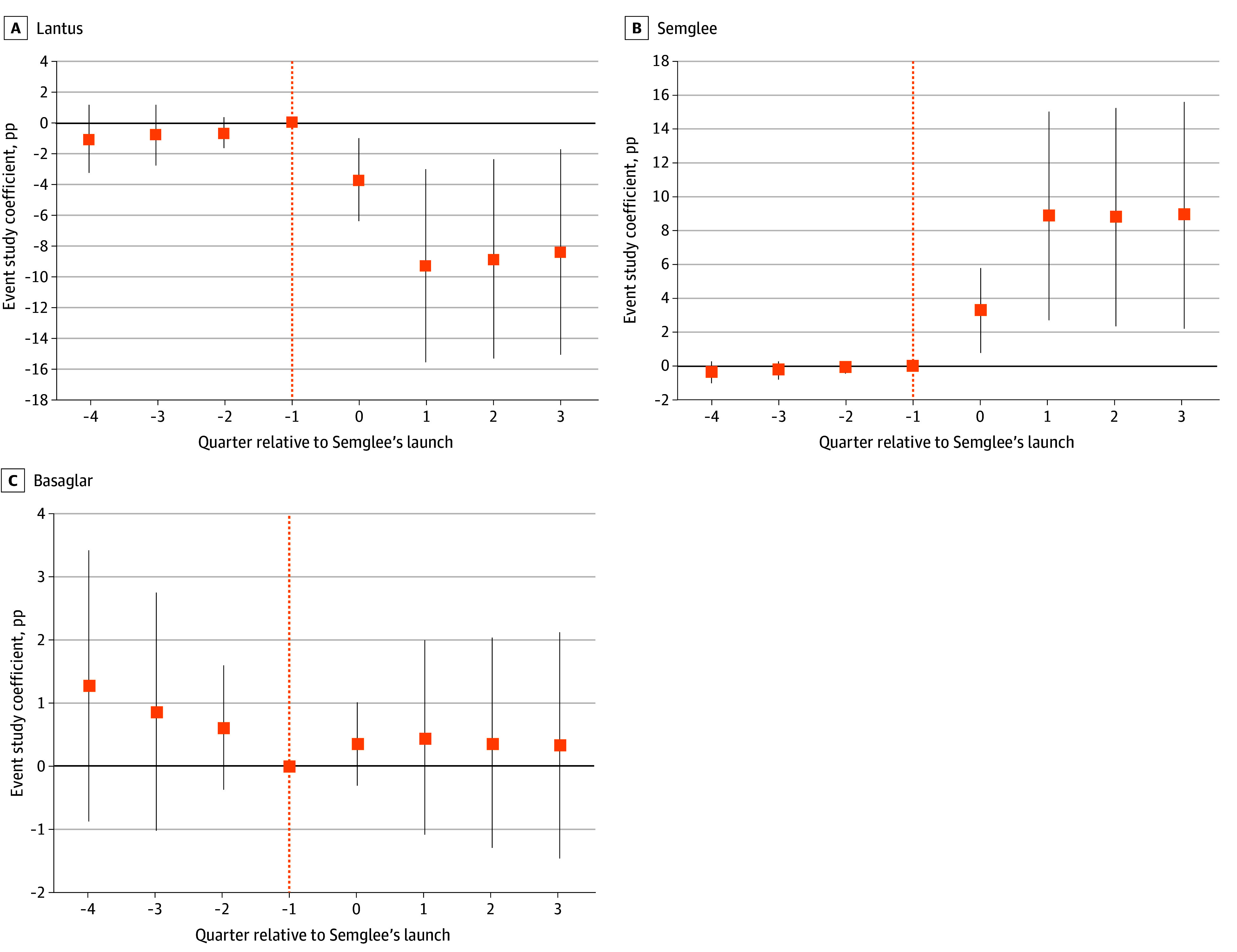
Event Study Estimates of Changes in Insulin Glargine Market Share Associated With State Substitution Laws The figure displays event study estimates (in percentage points [pp] with error bars indicating 95% CIs) showing differential changes in fills of a highly prescribed long-acting insulin glargine (Lantus [Sanofi]), interchangeable biosimilar insulin glargine-yfgn (Semglee [Mylan Pharmaceuticals]), and a noninterchangeable insulin glargine (Basaglar [Lilly]) between states with less restrictive substitution laws, relative to other states with more restrictive substitution laws. The denominator for each share is the total number of fills for the highly prescribed insulin glargine and its biosimilar products, interchangeable insulin glargine-yfgn and the noninterchangeable insulin glargine. The vertical dotted line in each panel corresponds to the reference quarter (the quarter immediately prior to the launch of interchangeable insulin glargine-yfgn on November 16, 2021). The event study model included person fixed effects and clustered standard errors at the state level.

## Discussion

We observed large differences in biosimilar adoption by state-level pharmacy substitution law restrictiveness. Enrollees residing in states with less restrictive laws were more likely to fill insulin glargine-yfgn, highlighting the role of substitution laws as an important determinant of biosimilar adoption. With many manufacturers pursuing interchangeability designations^10^ and calls to treat all biosimilars as interchangeable,^11^ reforms to substitution laws may be needed to clear undue barriers to substitution. One fruitful area of reform is revisiting the requirements regarding physician and patient notifications, given their ubiquity and pronounced associations with substitution.^12^ For example, waiving notification for refills or allowing a longer time frame for notification may lower the administrative burden for the substituting pharmacist.^13^

While reducing such regulatory hurdles may facilitate biosimilar adoption, increased substitution may not benefit all stakeholders equally. For patients, substitution may not translate into noticeable savings if plans prefer (and subsequently offer more favorable cost sharing for) reference products due to their higher list prices and rebates.^8,14^ However, increased substitution may foster biosimilar and reference product price competition, which may lower the net prices of drugs.^15,16^ These dynamics, along with other barriers to market entry,^17^ need to be more thoroughly explored as the biosimilar markets mature.

### Limitations

Although our model adjusted for time-fixed state confounders of biosimilar dispensing (via enrollee fixed effects), it may be biased by unmeasured factors (eg, local prescribing practices, awareness of biosimilars, patient preferences^1^) that may be time varying across states. Moreover, we observed the dispensing of biosimilar, not actual substitution practices. Furthermore, we lacked granular plan-level data that may impact dispensing. Finally, measurement errors in our characterization of state laws may have biased estimates.

## Conclusions

In this cohort study, state regulations of substitution were associated with differential uptake of an interchangeable long-acting insulin biosimilar product. Reforms to create a more favorable legal environment for substitution may contribute to the goal of expanding biosimilar use.

## Notes

Supplement 1.
eMethods
**eTable 1.** A list of NDC used for identifying Lantus, Semglee, and Basaglar fills **eFigure 1.** Distribution of state law summary score **eFigure 2.** A map of states by substitution law scores **eAppendix.** Robustness check results **eTable 2.** Difference-in-difference analysis estimating changes in fills for each type of long-acting insulin products **eTable 3.** Sensitivity of main treatment effect to excluding enrollees filling other types of long-acting insulin products **eFigure 3.** Results of “leave-one-out” analysis **eTable 4.** Sensitivity of main treatment effect to including the sample of enrollee-fills with imputed state-identifiers **eFigure 4.** Scenarios of “biased” treatment effects corresponding to imputation of treatment status to Lantus and biosimilar fills with missing state-identifiers **eTable 5.** Difference-in-difference analysis using alternate measures of exposure to estimate changes in Semglee fills **eTable 6.** Sensitivity to excluding each substitution law domain in calculating the main exposure variable
eReferences

Supplement 2.
Data Sharing Statement
