# Supplementary material for: State Substitution Laws and Uptake of an Interchangeable Insulin Biosimilar
Source: JAMA Health Forum. 2025 Apr 4;6(4):e250406. doi: 10.1001/jamahealthforum.2025.0406 (PMC11971668; doi:10.1001/jamahealthforum.2025.0406)
Supplement: Supplement 1. — eMethods eTable 1. A list of NDC used for identifying Lantus, Semglee, and Basaglar fills eFigure 1. Distribution of state law summary score eFigure 2. A map of states by substitution law scores eAppendix. Robustness check results eTable 2. Difference-in-difference analysis estimating changes in fills for each type of long-acting insulin products eTable 3. Sensitivity of main treatment effect to excluding enrollees filling other types of long-acting insulin products eFigure 3. Results of “leave-one-out” analysis eTable 4. Sensitivity of main treatment effect to including the sample of enrollee-fills with imputed state-identifiers eFigure 4. Scenarios of “biased” treatment effects corresponding to imputation of treatment status to Lantus and biosimilar fills with missing state-identifiers eTable 5. Difference-in-difference analysis using alternate measures of exposure to estimate changes in Semglee fills eTable 6. Sensitivity to excluding each substitution law domain in calculating the main exposure variable eReferences [file jamahealthforum-e250406-s001.pdf]

Supplemental Online Content

Kwon Y, Sarpatwari A, Dusetzina SB. State substitution laws and uptake of an interchangeable insulin biosimilar. *JAMA Health Form*. 2025;6(4):e250406. doi:10.1001/jamahealthforum.2025.0406

eMethods
eTable 1. A list of NDC used for identifying Lantus, Semglee, and Basaglar fills
eFigure 1. Distribution of state law summary score
eFigure 2. A map of states by substitution law scores
eAppendix. Robustness check results
eTable 2. Difference-in-difference analysis estimating changes in fills for each type of long-acting insulin products
eTable 3. Sensitivity of main treatment effect to excluding enrollees filling other types of long-acting insulin products
eFigure 3. Results of “leave-one-out” analysis
eTable 4. Sensitivity of main treatment effect to including the sample of enrollee-fills with imputed state-identifiers
eFigure 4. Scenarios of “biased” treatment effects corresponding to imputation of treatment status to Lantus and biosimilar fills with missing state-identifiers
eTable 5. Difference-in-difference analysis using alternate measures of exposure to estimate changes in Semglee fills
eTable 6. Sensitivity to excluding each substitution law domain in calculating the main exposure variable
eReferences

This supplemental material has been provided by the authors to give readers additional information about their work.

© 2025 Kwon Y et al. *JAMA Health Forum*.

eMethods.

Truven MarketScan Data

This retrospective longitudinal study analyzed Commercial Claims and Encounters (CCAE) data from the MarketScan databases by Truven Health Analytics.[1] The CCAE data contains medical claims of individuals, their dependents and spouses, who are covered by 1 of over 300 large-size employers that sponsor private health insurance and contribute data to the MarketScan. While not a nationally representative data, the CCAE contains claims for nearly ~200 million enrollees in the United States and is considered one of the largest administrative data for conducting health services research.[1,2] In this study, we used the outpatient prescription files, which contain actual prescriptions that are filled by the commercially insured enrollees captured in the database.

Constructing the study population

Our study cohort consisted of commercially insured enrollees, aged 1 to 64, who had at least one claim for Lantus, a long-acting insulin glargine,[3] or their biosimilars, including Semglee (insulin glargine-yfgn[4]) and Basaglar,[5] in one-year period before and after the date of interchangeable Semglee's launch on November 16, 2021. Thus, the cohort allowed us to observed changes in fills for Lantus and their biosimilars following Semglee's market launch among users of Lantus-equivalent insulin glargine products. In creating this cohort, we did not consider fills Toujeo, another branded long-acting insulin glargine product, [6,7] to meet the inclusion criteria, as Toujeo and Lantus are two different formulations of insulin glargine and Toujeo is not a biosimilar to Lantus. We identified fills for Lantus-equivalent insulin glargine products based on the National Drug Code (NDC) listed on each pharmacy claim (

© 2025 Kwon Y et al. *JAMA Health Forum*.

eTable **1**). In our data, the number of Semglee claims was low prior to its launch as an interchangeable product (<1% of market share), consistent with prior reports showing a sharp increase in the uptake of interchangeable Semglee following November of 2021.[8–10]

eTable 1. A list of NDC used for identifying Lantus, Semglee, and Basaglar fills

Product	NDC
Lantus	"50090087600", "54569560500", "68115083910", "55045368501", "54868462600", "54868576500", "49999099410", "00088222033", "00088222052", "00088502101", "54569646200", "54569646201", "68258893103", "54868623100", "00088222060", "50090139800", "50090406800", "00088221900", "00088221901", "00088221905", "00088502005"
Semglee	"49502039380", "83257001411", "49502039471", "49502039475", "83257001531", "83257001532", "50090583500", "49502019580", "49502025080", "83257001111", "49502019671", "49502019675", "49502025171", "49502025175", "83257001231", "83257001233"
Basaglar	"00002771501", "00002771559", "00002771563", "00002821405"

Abbreviation: NDC, National Drug Code

Characterizing state biosimilar substitution laws

We obtained a publicly available repository of state statutes and codes which detail legally permissive substitution of interchangeable biosimilar products from the National Association of Chain Drug Store (capturing state laws as of July, 2021).[11] The repository contains specific sections of each state's statue and code that list the definition of biologic/biosimilar products, circumstances when substitution is authorized, requirements for notifying prescribers or patients, and other legal considerations (e.g., labeling biosimilar drugs, potential liability for substitution, and requirements for recordkeeping). We ensured completeness of such information by supplementing the repository with online searches of state statues and codes in Google and free legal databases (e.g., Justia, FindLaw).

© 2025 Kwon Y et al. *JAMA Health Forum*.

Using the repository, we created seven binary variables, each equaling 1 if a state's substitution law included a requirement in the following domains:

- **Active approval required**: prescribers must expressly authorize substitution prior to substitution by pharmacists.
- **Enhanced physician notification**: Pharmacists must notify prescribing physicians and do so within <5 days of substitution (the 5-day cut-off was used because it was the most used cut-off for this requirement).
- **Notification for refills**: No clear language that exempts physician notification requirements for refills.
- **Patient notification**: Patients must be notified at the time of dispensing substituted medications.
- **Enhanced labeling requirement**: substitution must be explicitly indicated on labels using a language such as "substituted for" on the label.
- **Requirement for record retention**: Pharmacists must retain records of substitution for any number of durations.
- **Unspecified legal liability**: there is no clear language states that pharmacists assume no greater liability for substituting with biosimilar vs. the originator product.

These domains were selected because they circumscribe potentially more restrictive requirement on pharmacist discretion to substitute.[12]

Upon coding each state's substitution law, we sum the values of the substitution law variables, creating a summary score for each state, with higher scores denoting more "restrictions" on pharmacists who wish to substitute. The distribution of the summary score is shown in eFigure 1. We categorized states with a score of 3 or less (the median score) as

© 2025 Kwon Y et al. *JAMA Health Forum*.

representing states with less restrictive legal environment for pharmacist-level substitution (N=37). A state map of substitution law scores is available in eFigure 2, which shows that most variation in treatment occurs in the northeastern and southeastern regions of the United States. A full table of the state laws we coded is available in the following Github repository: https://github.com/yok84/BiosimilarSubstituitonLaws/blob/main/BiosimilarSubstitutionLaw_Online%20Supplement%202_Final_10242024.xlsx)

© 2025 Kwon Y et al. *JAMA Health Forum*.

eFigure 1. Distribution of state law summary score

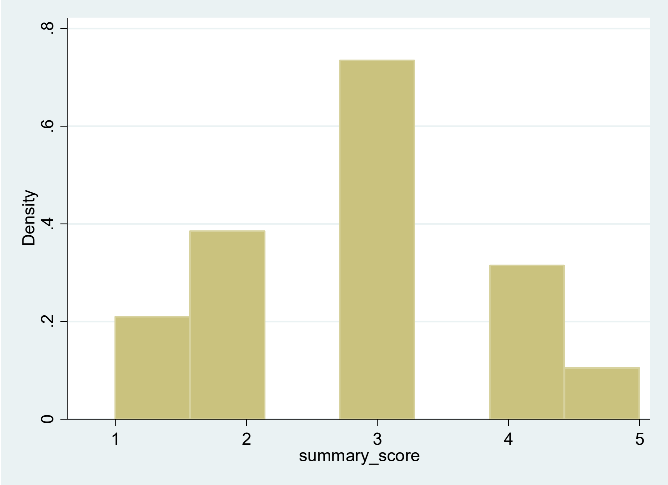

Note: The figure displays the histogram of the summary state law substitution score, which summarizes whether a state had any restriction on interchangeable biosimilar substitution in each domain of state laws: 1) Active prescriber approval required, 2) enhanced physician notification required (i.e., must notify prescribers within <5 days of substitution), 3) physician notification required for refills, 4) requirement for patient notification at the time of dispensing, 5) enhanced labeling requirement (i.e., the label must explicitly contain a language describing substitution, such as "substituted for"), 6) requirement for pharmacists to retain records of substitution, and 7) unclear legal liability for substituting with biosimilars. The mean and median scores were 2.64 and 3, respectively.

eFigure 2. A map of states by substitution law scores

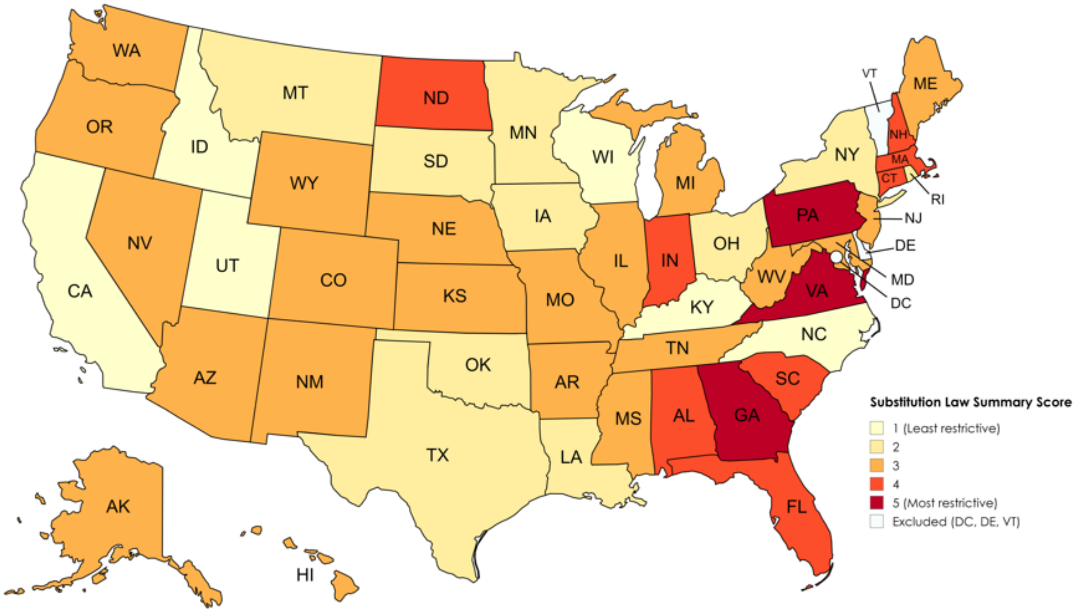

Note: The figure displays a map of states based on the summary state substitution law scores. States with a score of 3 or less (the median score) were categorized as less restrictive states, as they are more likely to feature more permissive legal environment for pharmacists who wish to substitute interchangeable biosimilar products. The map was created using mapchart.net (https://www.mapchart.net/). Less restrictive states are: Hawaii, Idaho, Vermont, Wyoming, Alaska, Montana, Rhode Island, South Dakota, New Mexico, Maine, Arkansas, Nebraska, West Virgina, Nevada, Mississippi, Oklahoma, Utah, Iowa, Louisiana, Colorado, Wisconsin, Minnesota, Michigan, Oregon, Kansas, Maryland, Arizona, Tennessee, Kentucky, New Jersey, Washington, Illinois, Missouri, North Carolina, New York, Ohio, California, and Texas. More restrictive states are: Alabama, Connecticut, Delaware, Florida, Georgia, Indiana, Massachusetts, New Hampshire, North Dakot, Pennsylvania, South Carol, and Virginia.

© 2025 Kwon Y et al. *JAMA Health Forum*.

Identification strategy: Difference-in-Differences analysis

Our analytic approach leverages both *between*-state variation in biosimilar substitution laws and *within*-person variation in exposure to Semglee (before and after its launch) to estimate effects of state substitution laws on utilization of biosimilars vs. branded biologic drugs. We fit the following linear probability difference-in-difference (DD) model:

$$y_{icst} = \alpha + \beta_1 Treated_{cs} + \beta_2 Post_{ct} + \beta_3 Treated_{cs} * Post_{ct} + \theta_i + \gamma_t + \varepsilon_{ist}$$

Where i indexes an enrollee, c indexes a pharmacy-claim, s indexes a state of dispensing, and t indexes a year-quarter. y_{icst} is the outcome (i.e. probability that the fill is for Lantus vs. non-Lantus products and Semglee vs. non-Semglee products). $Treated_{cs}$ is 1 for pharmacy claims that are filled in treated states (i.e., states with more "permissive" environment for substitution). $Post_{ct}$ is 1 for claims that are filled after Semglee's launch (November 16, 2021). β_3 is the DD coefficient showing differential changes in the outcome between pharmacy claims in treated and control states, before and after Semglee's launch. θ_i denotes enrollee fixed effects, which adjusts for time-invariant characteristics of an enrollee that may be correlated with the outcome. γ_t are year-quarter fixed effects, which adjusts for secular trend in the outcome. We clustered standard errors at the state-level, rather than at the enrollee-level, to reflect the fact that the treatment varies by state.

We also fit an event study form of DD to estimate dynamic treatment effects and test of parallel outcome trends between treated and control states in the pre-period:

$$y_{icst} = \alpha + \beta_1 Treated_{cs} + \sum_{\tau=-4,\tau\neq-1}^{3} \beta_\tau Treated_{cs} * \mathrm{I}[\tau = t]_{ct} + \theta_i + \gamma_t + \varepsilon_{ist}$$

where the post-indicator is replaced by a series of year-quarter dummies, $\mathrm{I}[\tau = t]_{ct}$, taking the year-quarter immediately before the launch date of Semglee as the reference period ($\tau = -1$). We

© 2025 Kwon Y et al. *JAMA Health Forum*.

test whether the event study coefficients for $\tau = -4, -3, -2$ are statistically indistinguishable from 0, which would suggest no significant pre-trends in outcomes.

Robustness checks

We conducted several sets of robustness checks to examine potential biases to our DD analysis. We conducted a falsification test by investigating differential changes in fills for Basaglar between treated and control states, before and after treatment. This exploits the fact that Basaglar was approved as a follow-on biologic drug under the Section 505(b) of the Food, Drug, and Cosmetic Act, not as an interchangeable biosimilar product,[13] and that all state substitution laws *only* permit automatic substitution with biosimilar products that receive the interchangeability designation by the FDA.[14] While Basaglar is legally a reference product, in essence, it is treated as a biosimilar as it relied on the evidence of safety and effectiveness of Lantus during its approval.[5,13,15] The falsification test would help us rule out potential unmeasured time-varying confounders, such as propensity for providers to *prescribe* any biosimilar products, that may be differential between treated and control states. A null finding in the falsification test would strengthen the validity of our main finding regarding the effect of state legal environment for substitution.

Moreover, we assessed patterns of switching *between* long-acting insulin products to ensure that lower Lantus fills we observed in treated states is driven by substitution *within* Lantus-equivalent products, rather than switch *from* Lantus *to* other types of long-acting insulin, Levemir (insulin detemir), Toujeo (insulin glargine), Tresiba (insulin degludec). To do so, we expanded our cohort to include all commercially insured enrollees who had fills for any long-acting insulin, not just Lantus-equivalent products, and replicated our DD analysis estimating differential changes in the probability of filling each type of long-acting insulin, pr(Levemir=1),

© 2025 Kwon Y et al. *JAMA Health Forum*.

pr(Toujeo=1), pr(Tresiba=1), and pr(Lantus-equivalent=1), between treated and control states. In addition, we repeated our main analysis but restrict the sample to *only* include enrollees who did not have fills for other types of long-acting insulin (i.e., stable users of Lantus-equivalent products throughout the study period).

Furthermore, we gauge the extent to which exclusions of fills with unknown state-identifiers may have biased our analysis. Recall that about a quarter of our initial sample (N=170,296) was excluded because their geographic identifiers only listed the Census region, rather than the state, where the fill was dispensed. Therefore, differences in the prevalence of fills with missing state-identifiers between treated and control states are potential sources of bias, though it is unclear, *ex ante*, whether they will bias our estimate towards or away from the null. For instance, we may have overestimated the treatment effect if fills for Lantus (biologic) were more likely to be excluded in treated states (where we hypothesize greater biologic-to-biosimilar substitutions) by artificially deflating the true number of biologic fills. The opposite would be the case if such fills were more likely to be excluded in control states. Thus, the direction and magnitude of the bias depends on two components: 1) the *prevalence* of missingness between treated and control states and 2) the *distribution* of Lantus vs. non-Lantus fills among excluded claims. It is impossible to directly calculate the first component, given that there is no *a priori* knowledge of the states in which the fill was actually dispensed. However, the second component *is* observed in the data, which makes it feasible to model a range of "biased" scenarios under which we can manipulate the distribution of Lantus vs. non-Lantus claims with unknown state identifiers between treated and control states.

Based on this framework, we performed two sets of sensitivity analyses. First, we utilized a simple imputation procedure, randomly assigning a state to fills with missing state-identifiers

© 2025 Kwon Y et al. *JAMA Health Forum*.

(but listing the Census region) according to the sampling distribution of states within each Census region. This procedure makes a strong assumption that the prevalence of missingness is random and proportional to the sample size in each state. In a combined sample containing the fills with imputed state identifiers, we estimate our main model, but additionally adjust for an indicator variable that is 1 for imputed fills.

Second, we conducted a scenario analysis that can more granularly model the second component in our bias framework. In this analysis, we used a modified imputation technique in which we assign the treatment indicator to fills with missing state-identifiers according to two parameters:

α = proportion of non-Lantus (biosimilar) fills to treated states

β = proportion of Lantus (biologic) fills to treated states.

For example, an imputation of α=100% and β=0% would model the "most biased" scenario in which all biosimilar fills would be assigned *treatment* and all fills for biologic fills would be assigned *controls*, and thus the rate of biologic-to-biosimilar switch is manipulated to be higher in treated states where we expect more permissive legal environment for substitution. At the opposite extreme, an imputation of α=0% and β=100% would underestimate the rate of biologic-to-biosimilar in treated states. After assigning treatment in this fashion, we then impute a state identifier based on the sampling distribution of fills in treated vs. control states within each Census region, as we have done for the first analysis. We then estimated our main DD model in the imputed sample. We essentially ran the analysis 121 times, modeling scenarios using imputation values of $0 \leq \alpha \leq 100\%$ and $0 \leq \beta \leq 100\%$ in a 10% increment (11 x 11 = 121).

We also tested our main model to using alternate measures of the treatment variable that characterized treatment using a simple binary variable that is 1 if a state had a requirement

© 2025 Kwon Y et al. *JAMA Health Forum*.

falling into one of the seven domains of substitution laws we measured. These alternate measures help us illuminate specific substitution law domains that may have more pronounced impacts on rates of biologic-to-biosimilar substitution. They also provide a way to hedge against potential misspecification of the exposure in our main analysis, which relied on the state law summary score to distinguish treated vs. control states. Additionally, we examined the sensitivity of our exposure variable to sequentially excluding each substitution law domain in our calculations.

Lastly, we conducted a “leave-one-out” analysis in which we iterate our analysis 50 times but leave out all observations from one state out of the analysis. The analysis can help identify potential influential states that may be disproportionately contributing to our estimate.

© 2025 Kwon Y et al. *JAMA Health Forum*.

eAppendix. Robustness check results

Falsification test using changes in Basaglar fills as outcome

In our falsification test, we did not detect significant differential changes in the probability of filling Basaglar between less vs. more restrictive states, before and after Semglee's launch (DD: -0.24 percentage-points, 95% CI: -1.40, 0.92, *P* = 0.675), as we had hypothesized (Table 2 in main text). Figure 2 in main text reports the event study coefficients, showing differential changes in the probability of filling Basaglar in each year-quarter, which suggest similar changes in the probability of filling Basaglar between two set of states *throughout* the study period.

Examining patterns of switching between long-acting insulin products

In a combined sample of enrollee-fills for all four types of long-acting insulin products, we found no significant changes in the probability of filling Levemir (DD: 0.24 percentage-points, 95% CI: -0.17, 0.65, *P* = 0.247), Tresiba (DD: 0.37 percentage-points, 95% CI: -0.27, 1.01, *P* = 0.256), or Toujeo (DD: -0.09 percentage-points, 95% CI: -0.53, 0.4=34, *P* = 0.669), between states with less vs. more restrictive laws, before and after Semglee's launch (eTable 2). We found a precisely estimated null effect on the probability of filling Lantus, Semglee, or Basaglar insulin glargine products (DD: -0.61 percentage-points, 95% CI: -1.40, 0.18, *P* =0.128).

© 2025 Kwon Y et al. *JAMA Health Forum*.

eTable 2. Difference-in-difference analysis estimating changes in fills for each type of long-acting insulin products

	Outcome			
	**Pr(Lantus, Semglee, or Basglar=1)**	**Pr(Levemir=1)**	**Pr(Tresiba=1)**	**Pr(Toujeo=1)**
DD in pp (95% CI)	-0.61 (-1.40, 0.18)	0.24 (-0.17, 0.65)	0.37 (-0.27, 1.01)	-0.09 (-0.53, 0.34)
P-value	0.128	0.247	0.256	0.669
Pre-mean, treated	61.38	14.32	24.30	11.23
% Change	-1.1	2.1	1.6	-0.8
Enrollee FE	Yes	Yes	Yes	Yes
Year-quarter FE	Yes	Yes	Yes	Yes
N	1,025,184	1,025,184	1,025,184	1,025,184

Abbreviations: DD, difference-in-differences; pp, percentage-points; CI, confidence interval; FE, fixed effects
Note: The table displays difference-in-differences coefficients (and their 95% confidence intervals) estimating differential changes in the probability of filling each type of long-acting insulin products (i.e., Lantus and its biosimilars , Levemir, Tresiba, or Toujeo) in a larger sample of enrollee-fills for long-acting insulin products. The coefficients are shown as percentage-points. Our model adjusted for enrollee and year-quarter fixed effects and clustered standard errors at the state-level.

Moreover, our treatment effect is not sensitive to excluding enrollees who had fills for non-Lantus long-acting insulin products during the study period. eTable 3 reports two DD coefficients estimated among the original sample and the refined sample that excludes "switchers", and they are virtually identical. These results suggest that the patterns of switching between long-acting insulin products were similar between treated and control states over time and our main treatment effect is likely driven by substitution *within* Lantus with its biosimilars.

eTable 3. Sensitivity of main treatment effect to excluding enrollees filling other types of long-acting insulin products

	**(1) Original sample**	**(2) "No switcher" sample**
DD in pp (95% CI)	-6.48 (-11.70,-1.26)	-6.83 (-11.98, -1.67)
P-value	0.016	0.010
Pre-mean, treated	59.56	59.28
% Change	-11.1	-11.5
Enrollee FE	Yes	Yes
Year-quarter FE	Yes	Yes
N	487,281	322,602

Abbreviations: DD, difference-in-differences; pp, percentage-points; CI, confidence interval; FE, fixed effects
Note: The table compares two difference-in-differences coefficients (and their 95% confidence intervals) estimating differential changes in the probability of filling Lantus vs. biosimilars among 1) the original sample and 2) the refined sample that excludes enrollees who had fills for non-Lantus long-acting insulin products (Levemir, Tresiba, or Toujeo) throughout the study period (i.e., stable users of Lantus or its biosimilar products). The coefficients are shown as percentage-points. Our model adjusted for enrollee and year-quarter fixed effects and clustered standard errors at the state-level.

© 2025 Kwon Y et al. *JAMA Health Forum*.

Leave-one-out analysis

Results from leave-one-out analysis indicate no influential or outlier states in our data (eFigure 3). All DD coefficients estimated in each iteration of analysis were significant (*P* < .05) and they are clustered tightly around the main treatment effect (-6.48 percentage-points).

eFigure 3. Results of “leave-one-out” analysis

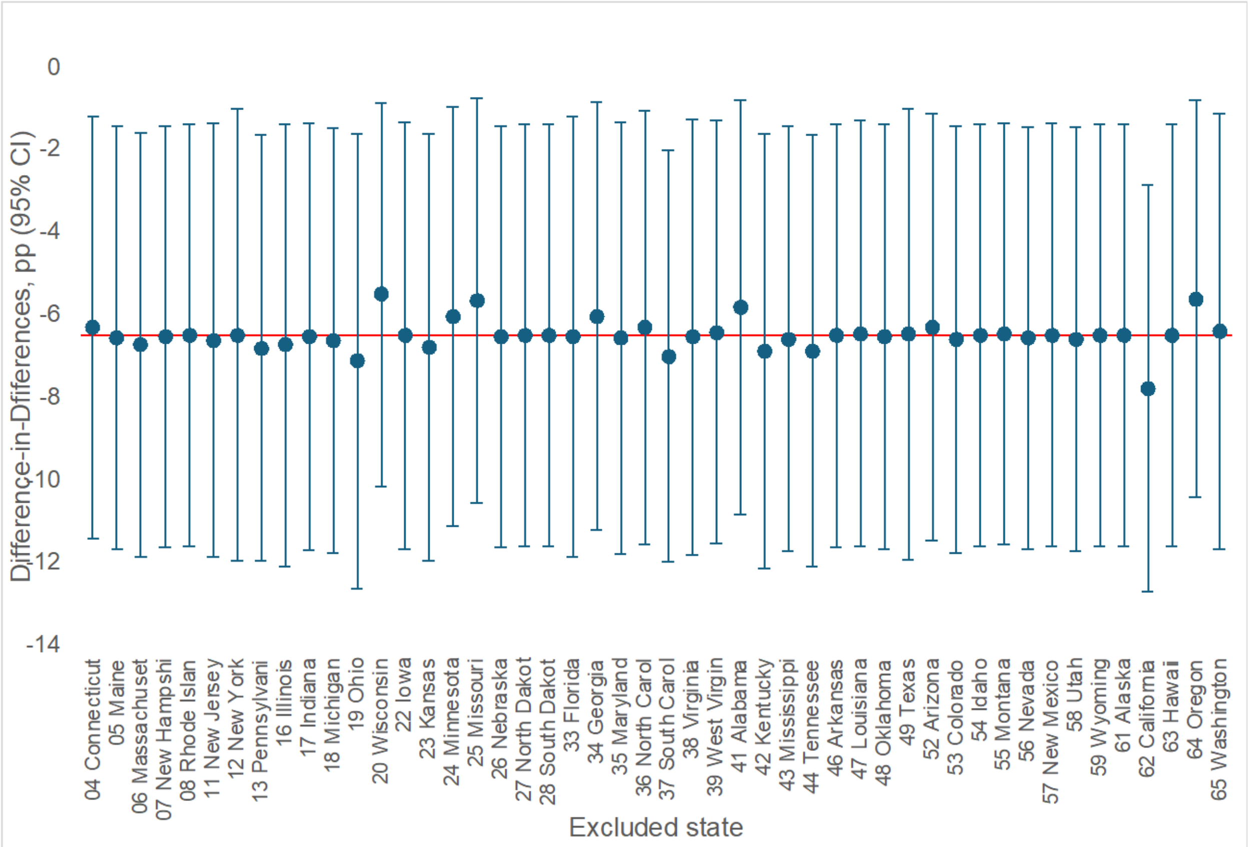

Abbreviations: pp, percentage-points; CI, confidence interval
Note: Each scatter-point corresponds to DD coefficient (and its 95% confidence interval) from each iteration of “leave-one-out” analysis that excluded all observations from the listed state in the X-axis. The horizontal red-line equates to the treatment effect from the main analysis (-6.48 percentage-points). All models adjusted for enrollee- and year-quarter fixed effects and clustered standard errors at the state-level.

© 2025 Kwon Y et al. *JAMA Health Forum*.

Assessment of bias arising from excluding fills with missing state-identifiers

In eTable 4, we report the DD coefficient estimated among the sample that includes enrollee-fills for which we randomly assigned state-identifiers within the Census region of dispensing. In Column (2), we show that the DD estimate in this sample was smaller in magnitude compared to the main DD estimate (-4.42 percentage-points, 95% CI: -10.44, 1.60, *P* = 0.146), though their confidence intervals overlap and are statistically indistinguishable. We note that the coefficient for the "imputed" indicator was not significant (beta: 2.10, 95% CI: -2.66, 6.87, *P* = 0.380), which does suggest that missing state-identifiers, at least in this context, is not likely associated with the outcome.

eTable 4. Sensitivity of main treatment effect to including the sample of enrollee-fills with imputed state-identifiers

	**(1)**<br>**Original sample**	**(2)**<br>**Original +**<br>**Imputed sample**
DD in pp (95% CI)	-6.48 (-15.6, -2.3)	-4.42 (-10.44, 1.60)
P-value	0.009	0.146
β, imputed indicator (95% CI)	-	2.10 (-2.66, 6.87)
P-value	-	0.380
Pre-mean, treated	59.56	59.7
% Change	-15.5	9.7
Enrollee FE	Yes	Yes
Year-quarter FE	Yes	Yes
N	487,281	637,136

Abbreviations: DD, difference-in-differences; pp, percentage-points; CI, confidence interval; FE, fixed effects
Note: The table compares two difference-in-differences coefficients (and their 95% confidence intervals) estimating differential changes in the probability of filling Lantus vs. biosimilars among 1) the original sample and 2) the sample that includes enrollee-fills with imputed state-identifiers. For fills with missing state-identifiers, we randomly assigned states based on the sampling distribution of states within the Census region that was listed in the geographic identifier. The coefficients are shown as percentage-points. Our model adjusted for enrollee and year-quarter fixed effects and clustered standard errors at the state-level. In Column 2, we also report the regression coefficient for the variable indicating imputation, which was additionally adjusted as a covariate in the model.

In our scenario analysis, our estimates of treatment effects ranged from -13.37 percentage-points ($P < 0.001$) when α=100% and β=0% to 4.75 percentage-points (not significant) when α=0% and β=100% (eFigure 4). The direction of treatment effect was negative

© 2025 Kwon Y et al. *JAMA Health Forum*.

in 85% (N=103) and significantly negative in 50% (N=61) of 121 modeled scenarios. Furthermore, positive treatment effects (none was significant) were concentrated in the right upper corner region, representing areas in which we expect more extreme values of α and β. These results establish a high threshold for biases associated with excluding fills with missing state-identifiers, though we cannot *conclusively* rule out such biases given that we do not actually observe the true location of dispensing for these fills. However, the preponderance of scenarios with negative treatment effects increases the confidence that excluding these fills would not have appreciably impacted our main analysis.

eFigure 4. Scenarios of "biased" treatment effects corresponding to imputation of treatment status to Lantus and biosimilar fills with missing state-identifiers

α \ β	0%	10%	20%	30%	40%	50%	60%	70%	80%	90%	100%
0%	-3.28	-2.55	-1.85	-1.14	-0.43	0.32	1.09	1.88	2.72	3.66	4.75
10%	-4.16	-3.44	-2.74	-2.04	-1.33	-0.59	0.17	0.96	1.79	2.70	3.69
20%	-5.07*	-4.35*	-3.66	-2.95	-2.25	-1.51	-0.76	0.02	0.84	1.74	2.70
30%	-5.98*	-5.27*	-4.58*	-3.88	-3.18	-2.45	-1.70	-0.93	-0.12	0.77	1.71
40%	-6.90**	-6.19**	-5.50*	-4.80*	-4.11*	-3.38	-2.64	-1.88	-1.08	-0.20	0.73
50%	-7.89**	-7.18**	-6.49**	-5.80**	-5.11*	-4.39*	-3.66	-2.90	-2.12	-1.25	-0.34
60%	-8.86***	-8.15**	-7.46**	-6.77**	-6.09**	-5.37*	-4.65*	-3.91	-3.13	-2.28	-1.38
70%	-9.92***	-9.22***	-8.53***	-7.85**	-7.17**	-6.46**	-5.75**	-5.02*	-4.26	-3.43	-2.55
80%	-10.99***	-10.28***	-9.60***	-8.92***	-8.25**	-7.55**	-6.85**	-6.14*	-5.40*	-4.58	-3.72
90%	-12.18***	-11.46***	-10.78***	-10.11**	-9.45**	-8.76**	-8.08**	-7.38**	-6.66*	-5.87*	-5.02
100%	-13.37***	-12.63***	-11.95**	-11.28**	-10.63**	-9.96**	-9.29**	-8.62**	-7.92*	-7.15*	-6.26*

Legend
- Positive treatment effect, not significant
- Negative treatment effect, not significant
- Negative treatment effect, P < 0.05
- Negative treatment effect, P < 0.01
- Negative treatment effect, P < 0.001

Note: Each cell in the table displays the DD coefficient that was estimated among the sample that includes enrollee-fills for which we impute treatment status based on two parameters, α (the proportion of biosimilar fills with missing state identifiers assigned to treated states), and β (the proportion of Lantus fills with missing state identifiers assigned to control states). For example, α=100% and β=0% indicate an assignment of treatment where all biosimilar fills with missing state-identifiers are assigned treatment and all Lantus fills with missing state-identifiers are assigned controls. After assigning treatment status, we impute state-identifiers based on the distribution of treated vs. control states within the Census region that was listed as the location of dispensing. Cells shaded in orange (blue) indicate a scenario where the treatment effect was negative (positive). We denote statistically significant treatment effects using darker shades (see legend).

© 2025 Kwon Y et al. *JAMA Health Forum*.

Results of difference-in-difference analysis using alternate treatment measures

eTable 5 reports seven DD coefficients, each estimated using an alternative treatment variable that tests for the effect of the specific domain of state substitution laws. We found more pronounced effects of requirement for enhanced physician notification (DD: -10.8, 95% CI: -16.5, -5.2, *P* < 0.001), notification requirement for refills (DD: -6.0, 95% CI: -11.1, -0.8, *P* = 0.024), and patient notification (DD: -4.5, 95% CI: -10.9, 1.9, *P* = 0.165) on uptake of Semglee in the post-period. All other treatment variables, except the record retention variable, was associated with lower uptake of Semglee, though we cannot rule out large effects in either direction.

eTable 5. Difference-in-difference analysis using alternate measures of exposure to estimate changes in Semglee fills

	**Pr(Semglee=1)**		
**Exposure variable**	**DD in pp**	**95% CI**	***P*-value**
Active approval	0.54	-7.30, 8.38	0.891
Enhanced physician notification	-8.15	-12.49, -3.81	< 0.001
Notification for refills	-4.68	-8.78, -0.58	0.026
Patient notification	-3.52	-8.44, 1.40	0.157
Enhanced labeling	-0.01	-6.77, 6.75	0.997
Record retention	-0.98	-7.94, 5.98	0.779
Unspecified legal liability	-3.16	-10.33, 4.02	0.381

Abbreviations: DD, difference-in-differences; pp, percentage points; CI, confidence interval; Pr, probability
Note: Each row displays a separate DD coefficient (and their 95% confidence intervals and *P*-values) showing differential changes in the probability of filling Semglee, which was estimated using an alternate treatment variable that equals 1 if a state had a requirement for each listed domain of substitution law. Our models adjusted for enrollee- and year-quarter fixed effects and clustered standard errors at the state-level.

In a separate sensitivity analysis, we systematically removed each substitution law domain in our calculation of summary law score for each state and re-characterizing the exposure. Our results were robust in the analysis, as our DD estimates are in similar ranges across each excluded scenario.

© 2025 Kwon Y et al. *JAMA Health Forum*.

eTable 6. Sensitivity to excluding each substitution law domain in calculating the main exposure variable

Excluded domain	Adjusted difference-in-differences	95% CI	P-value
Active approval	-5.61	-10.30, -0.91	0.02
Physician notification required within <5 days	-4.76	-9.73, 0.21	0.06
Notification required for refills	-6.11	-11.25, -0.97	0.021
Patient notification required	-6.39	-11.7, -1.03	0.021
Enhanced labeling requirement	-6.91	-12.27, -1.54	0.013
Requirement for record retention	-3.79	-10.30, 2.73	0.248
Unspecified legal liability	-3.02	-9.72, 3.67	0.369

Abbreviations: DD, difference-in-differences; pp, percentage points; CI, confidence interval; Pr, probability
Note: Each row displays a separate DD coefficient (showing differential changes in the probability of filling Lantus) that estimated using an alternate exposure variable which excluded each listed domain when calculating the summary state law score. Our models adjusted for enrollee- and year-quarter fixed effects and clustered standard errors at the state-level.

© 2025 Kwon Y et al. *JAMA Health Forum*.

eReferences

1. Kulaylat AS, Schaefer EW, Messaris E, Hollenbeak CS. Truven Health Analytics MarketScan Databases for Clinical Research in Colon and Rectal Surgery. *Clin Colon Rectal Surg*. 2019;32(1):54-60. doi:10.1055/s-0038-1673354

2. Rassen JA, Bartels DB, Schneeweiss S, Patrick AR, Murk W. Measuring prevalence and incidence of chronic conditions in claims and electronic health record databases. *Clinical Epidemiology*. 2018;11:1-15. doi:10.2147/CLEP.S181242

3. Owens, Griffiths S. Insulin Glargine (lantus®). *International Journal of Clinical Practice*. 2002;56(6):460-466. doi:10.1111/j.1742-1241.2002.tb11297.x

4. The First Interchangeable Biosimilar Insulin: Insulin Glargine-yfgn - Mark C. Matli, Andrea B. Wilson, Leah M. Rappsilber, Farron P. Sheffield, Miranda L. Farlow, Jeremy L. Johnson, 2023. Accessed October 1, 2024. https://journals.sagepub.com/doi/full/10.1177/19322968211067511

5. Newsome C. Basaglar. *Clin Diabetes*. 2017;35(3):181. doi:10.2337/cd017-0025

6. Grant JS, Graven LJ. Insulin Glargine U-300 (Toujeo®): A Review. *Home Healthcare Now*. 2016;34(3):156. doi:10.1097/NHH.0000000000000361

7. Toujeo vs. Lantus: 6 Ways They're Different. GoodRx. Accessed October 1, 2024. https://www.goodrx.com/conditions/diabetes-type-1/toujeo-vs-lantus

8. Biosimilars Council. Semglee Launch Tracking. Published online July 2023. Accessed January 14, 2025. https://biosimilarscouncil.org/wp-content/uploads/2023/07/Biosimilars-Council-Semglee-Launch-Tracking-2023.pdf

9. Schroll M, Bizar-Brody O, Peake A, Felcsuti B. The Humira Biosimilars Have Arrived! Will They Make a Difference? Published online February 22, 2023. Accessed January 14, 2025. https://healthadvances.com/insights/blog/the-humira-biosimilars-have-arrived-will-they-make-a-difference

10. Samsung Bioepis. Samsung Bioepis Biosimilar Market Report, 4th Edition, Q1 2024. Published online 2024. Accessed January 14, 2025. https://www.samsungbioepis.com/upload/attach/SB+Biosimilar+Market+Report+Q1+2024.pdf

11. National Association of Chain Drug Store. State Substitution Practices for Biological Drugs. Published online July 2021. doi:https://www.nacds.org/pdfs/government/2021/State-Substitution-Practices-for-Biological-Drugs-chart-July-2021.pdf

12. Benedict AL. State-level legislation on follow-on biologic substitution. *Journal of Law and the Biosciences*. 2014;1(2):190-201. doi:10.1093/jlb/lsu005

13. Camlyn Masuda P, Rosalind Wong PC, Chase Ibia PC. Common Patient Questions About Insulin Biosimilars Pertain to Classification. 2023;12. Accessed October 2, 2024. https://www.pharmacytimes.com/view/common-patient-questions-about-insulin-biosimilars-pertain-to-classification

14. Gabay M. Biosimilar Substitution Laws. *Hosp Pharm*. 2017;52(8):544-545. doi:10.1177/0018578717726995

15. Mouslim M, Rashidi E, Levy J, Socal M, Trujillo A. The Price Paradox of Biosimilar-Like Long-Acting Insulin. 2022;28. Accessed September 30, 2024. https://www.ajmc.com/view/the-price-paradox-of-biosimilar-like-long-acting-insulin

© 2025 Kwon Y et al. *JAMA Health Forum*.
