# Supplementary material for: State Substitution Laws and Uptake of an Interchangeable Insulin Biosimilar
Source: JAMA Health Forum. 2025 Apr 4;6(4):e250406. doi: 10.1001/jamahealthforum.2025.0406 (PMC11971668; doi:10.1001/jamahealthforum.2025.0406)
Supplement: Supplement 2. — Data Sharing Statement [file jamahealthforum-e250406-s002.pdf]

Data Sharing Statement

Kwon. State Substitution Laws and Uptake of an Interchangeable Insulin Biosimilar. *JAMA Health Forum*. Published April 04, 2025. doi:10.1001/jamahealthforum.2025.0406

Data
Data available: No

Additional Information
Explanation for why data not available: We analyzed the MarketScan data that was made available through a data use agreement with our institution, and thus it cannot be shared.
